# Polychemotherapy with Curcumin and Doxorubicin via Biological Nanoplatforms: Enhancing Antitumor Activity

**DOI:** 10.3390/pharmaceutics12111084

**Published:** 2020-11-11

**Authors:** Milad Ashrafizadeh, Ali Zarrabi, Farid Hashemi, Amirhossein Zabolian, Hossein Saleki, Morteza Bagherian, Negar Azami, Atefe Kazemzade Bejandi, Kiavash Hushmandi, Hui Li Ang, Pooyan Makvandi, Haroon Khan, Alan Prem Kumar

**Affiliations:** 1 Faculty of Engineering and Natural Sciences, Sabanci University, Orta Mahalle, Üniversite Caddesi No. 27, Orhanlı, Tuzla, 34956 Istanbul, Turkey; milad.ashrafizadeh@sabanciuniv.edu; 2 Sabanci University Nanotechnology Research and Application Center (SUNUM), Tuzla, 34956, Istanbul, Turkey; alizarrabi@sabanciuniv.edu; 3 Department of Comparative Biosciences, Faculty of Veterinary Medicine, University of Tehran, Tehran 1419963114, Iran; faridhashemi172@gmail.com; 4 Young Researchers and Elite Club, Tehran Medical Sciences, Islamic Azad University, Tehran 1916893813, Iran; ah_zabolian@student.iautmu.ac.ir (A.Z.); h.saleki@student.iautmu.ac.ir (H.S.); morteza1429@gmail.com (M.B.); negarazami77@gmail.com (N.A.); atefe.kazemzade1376@gmail.com (A.K.B.); 5 Department of Food Hygiene and Quality Control, Division of Epidemiology & Zoonoses, Faculty of Veterinary Medicine, University of Tehran, Tehran 1419963114, Iran; houshmandi.kia7@ut.ac.ir; 6 Cancer Science Institute of Singapore and Department of Pharmacology, Yong Loo Lin School of Medicine, National University of Singapore, Singapore 117599, Singapore; e0336095@u.nus.edu; 7 Centre for Micro-BioRobotics, Istituto Italiano di Tecnologia, viale Rinaldo Piaggio 34, 56,025 Pontedera, Pisa, Italy; 8 Department of Pharmacy, Abdul Wali Khan University, Mardan 23200, Pakistan; haroonkhan@awkum.edu.pk

**Keywords:** doxorubicin, curcumin, chemoresistance, side effect, apoptosis, nanodelivery

## Abstract

Doxorubicin (DOX) is a well-known chemotherapeutic agent extensively applied in the field of cancer therapy. However, similar to other chemotherapeutic agents such as cisplatin, paclitaxel, docetaxel, etoposide and oxaliplatin, cancer cells are able to obtain chemoresistance that limits DOX efficacy. In respect to dose-dependent side effect of DOX, enhancing its dosage is not recommended for effective cancer chemotherapy. Therefore, different strategies have been considered for reversing DOX resistance and diminishing its side effects. Phytochemical are potential candidates in this case due to their great pharmacological activities. Curcumin is a potential antitumor phytochemical isolated from *Curcuma longa* with capacity of suppressing cancer metastasis and proliferation and affecting molecular pathways. Experiments have demonstrated the potential of curcumin for inhibiting chemoresistance by downregulating oncogene pathways such as MMP-2, TGF-β, EMT, PI3K/Akt, NF-κB and AP-1. Furthermore, coadministration of curcumin and DOX potentiates apoptosis induction in cancer cells. In light of this, nanoplatforms have been employed for codelivery of curcumin and DOX. This results in promoting the bioavailability and internalization of the aforementioned active compounds in cancer cells and, consequently, enhancing their antitumor activity. Noteworthy, curcumin has been applied for reducing adverse effects of DOX on normal cells and tissues via reducing inflammation, oxidative stress and apoptosis. The current review highlights the anticancer mechanism, side effects and codelivery of curcumin and DOX via nanovehicles.

## 1. Introduction

The necessity for having a new approach in chemotherapy is due to an emerging phenomenon, known as “chemoresistance” [1]. Triggering apoptosis, DNA damage, mitotic catastrophe, impairing microtubule stabilization and so on are responsible for antitumor activity of chemotherapeutic agents. However, frequent utilization of a specific chemotherapeutic agent is correlated with resistance of cancer cells to apoptosis induction [2,3]. Furthermore, cancer cells can prevent DNA damage following chemotherapy by stimulating molecular pathways involved in DNA repair. Thus, it is quite obvious that a new regime should be recommended for effective chemotherapy of cancer cells.

Increasing evidence demonstrates that phytochemicals, due to their excellent antitumor activity, can be considered as potential agents for coadministration with chemotherapeutic agents in cancer therapy [4,5]. Phytochemicals impair proliferation and metastasis of cancer cells, and sensitize cancer cells to apoptosis and DNA damage caused by chemotherapeutic agents [6,7,8]. This polychemotherapy with natural compounds is of importance in reversing chemoresistance. Interestingly, chemoresistance is not the only challenge in the field of chemotherapy. In order to promote antitumor activity of chemotherapeutic agents, high doses of these agents are applied that substantially enhances side effects on other organs of body. Ototoxicity [9], hepatotoxicity [10], nephrotoxicity [11] and cardiotoxicity [12] are a few of adverse effects of chemotherapeutic agents.

Based on pharmacological activities of natural products such as antioxidant that is of importance for reducing levels of reactive oxygen species (ROS) and a subsequent decrease in side effects of chemotherapeutics, coadministration of phytochemical and chemotherapeutic agents not only promotes antitumor activity and reverses chemoresistance, but also is beneficial in improving side effects [13,14]. Further issue that can be addressed is the poor bioavailability of phytochemicals and chemotherapeutic agents. Nanoarchitectures are structures with a size of less than 100 nm that can be synthesized via different methods [15,16,17]. Nanoparticles can provide a platform for co-loading of plant derived-natural products and chemotherapeutic agents in effective cancer therapy [18,19].

In the present review, we discuss potential of curcumin as a natural product for being employed in combination chemotherapy with doxorubicin. Potentiating antitumor activity of doxorubicin against cancer cells, reversing chemoresistance and nanocarriers for codelivery of curcumin and doxorubicin are described in this review article based on newly published articles.

## 2. Curcumin: Structure and Pharmacokinetics

As a curcuminoid, curcumin is a bioactive component of rhizome of *Curcuma longa* and comprises 1.5–3 weight (wt)% of this plant [20]. Turmeric possesses three components including curcumin, bisdemethoxycurcumin and demethoxycurcumin. The most abundant component of turmeric is curcumin [21]. This yellow pigmented powder is derived from rhizome of turmeric via grinding [22]. It is responsible for the yellow color of turmeric and for a long time, this flavoring substance has been widely applied in different regions of Asia for treatment of various disorders [23,24,25,26]. The chemical formula of curcumin is *n*,1,7-bis-(3-hydroxy-4-methoxyphenyl)-1,6-heptadiene-3,5-dione. Structurally, curcumin has two phenyl rings substituted with hydroxyl and methoxyl groups connected via a seven-carbon keto-enol linker [27,28]. The first discovered pharmacological activity of curcumin is an antibacterial effect, found in 1949 [29]. Further studies focused on revealing other therapeutic and biological activities of curcumin including antiproliferative [30], antimetastatic [31], antioxidant [32], anti-inflammatory [33], antidiabetic [34], antiangiogenic [35], immunomodulatory [36], antitumor [37], antiatherosclerotic [38] and a lipid lowering effect [39] and regulating blood pressure [40].

However, rapid metabolization of curcumin in body is a major impediment towards its therapeutic effects [41]. Upon oral administration of curcumin (1 g/kg), as a result of first pass metabolism, it is rapidly metabolized and 75% of this valuable agent is eliminated in feces with partial amounts in urine. Up to 50% of curcumin is excreted in bile following intravenous administration [42]. The major metabolite of curcumin in body is curcumin glucuronide and other metabolites include curcumin sulfate, hydroxycurcumin, hexahydrocurcuminol and hexahydrocurcumin glucuronide. Curcumin is well-tolerated in humans. The concentration of curcumin reaches its peak 1–2 h after oral administration and begins a decrease after 12 h [43]. Oral administration of 8 g curcumin results in a serum concentration of up to 2 μM, showing its poor bioavailability. However, curcumin is completely safe in humans, so that its administration for 8 months led to no adverse effect in patients [44].

## 3. Antitumor Activity of Curcumin: In Vitro, In Vivo and Clinical Studies

As a well-known natural product with excellent pharmacological function, antitumor activity of curcumin has been investigated against several sorts of cancer cells in different phases including in vitro, in vivo and clinical studies. Noteworthy, all of the experiments are in agreement with the fact that curcumin is a potential agent in cancer therapy [30,45]. Curcumin can impair both proliferation and metastasis of cancer cells via inducing cell cycle arrest, triggering cytoskeletal remodeling and inhibiting epithelial-to-mesenchymal transition (EMT). In this way, molecular pathways such as Wnt and Shh that are involved in cancer progression, undergo downregulation upon curcumin administration [46]. The inhibitory effect of curcumin on viability and colony formation of cancer cells is vital for enhancing sensitivity of cancer cells into chemotherapy [47].

The interesting point is the effect of curcumin on transcription factors in cancer therapy. MicroRNAs (miRNAs) are able to regulate different biological processes in cells and their dysregulation is correlated with the development of cancer [48,49]. In suppressing metastasis of cancer cells, curcumin promotes expression of miRNA-34a, as an oncosuppressor to inhibit EMT [50]. In addition to affecting molecular pathways involved in cancer metastasis, curcumin possesses the capacity of targeting molecular pathways that are responsible for cancer growth and survival. Increasing evidence demonstrates association of phosphatidylinositol 3-kinase (PI3K)/protein kinase B (Akt) pathway with cancer growth and migration [51,52]. Curcumin induces apoptosis and cell cycle arrest at the G2/M phase via the downregulating PI3K/Akt signaling pathway [53]. In vivo experiments have also confirmed the efficacy of curcumin in exerting antiproliferative activity in tumor models [54,55].

It has been demonstrated that antitumor activity of curcumin is dose- and time-dependent [56]. Noteworthy, combination of curcumin and chemotherapeutic agents is recommended in clinical studies [57]. Using nanocarriers such as liposomes significantly promote antitumor activity of curcumin in cancer therapy and is well-tolerated [58]. Although we mentioned poor bioavailability of curcumin as a barrier for its therapeutic effects, a clinical study has demonstrated plasma levels of curcumin as much as 22–41 ng/mL for 4 weeks that is of importance in the treatment of cancer patients [59]. So, studies are in agreement with potential antitumor activity of curcumin against different cancers and capability of targeting molecular pathways [60,61,62].

## 4. Curcumin in Cancer Chemotherapy: Beyond Doxorubicin

Based on the biological activities of curcumin (e.g., antioxidant and anti-inflammatory, and along with antitumor activity) a large number of experiments have been conducted using curcumin in combination chemotherapy. This section deals with a brief explanation for using curcumin as an adjuvant to enhance antitumor activity and diminishing the side effects of chemotherapeutic agents.

Paclitaxel resistance is frequently observed in cancer therapy and is associated with poor prognosis. Nuclear factor-kappaB (NF-κB) is suggested to be involved in mediating paclitaxel resistance in hepatocellular carcinoma (HCC) via upregulating Lin28B. Curcumin administration (5, 10 and 20 μM) leads to suppressing NF-κB-mediated Lin28B and preventing paclitaxel resistance in HCC cells [63]. It is worth mentioning that curcumin is capable of affecting drug transporters in enhancing sensitivity of cancer cells to paclitaxel chemotherapy. In this way, curcumin reduces activity and expression of p-glycoprotein (P-gp) and multidrug resistance-1 (MDR1) to promote paclitaxel accumulation in cancer cells [64]. Nanotechnology can provide a platform for codelivery of curcumin and paclitaxel. This results in promoting the bioavailability and enhancing accumulation in the cargo in cancer cells [65,66]. This consequence is of importance in providing effective cancer chemotherapy. Curcumin is beneficial in the alleviation of side effects of cisplatin during chemotherapy. For instance, curcumin (200 mg/kg) can improve renal fibrosis during cisplatin chemotherapy downregulation of transforming growth factor-beta 1 (TGF-β1) [67].

Nuclear factor erythroid 2-related factor 2 (Nrf2) as a molecular pathway involved in reinforcing the antioxidant defense system [68] is upregulated by curcumin in reducing cisplatin-mediated nephrotoxicity [69]. In suppressing cisplatin resistance, curcumin upregulates expression of miRNA-497 to sensitize cancer cells to apoptosis [70]. Overall, studies show that curcumin is a potential agent for ameliorating side effects of chemotherapeutic agents and enhancing their cytotoxicity against cancer cells [71,72,73]. In the following section, we evaluated the potential application of curcumin in reversing doxorubicin resistance and reducing its adverse effects.

## 5. Doxorubicin: Cancer Resistance and Side Effects

Doxorubicin (DOX) is a member of anthracycline antibiotic family derived from fungus *Streptomyces peucetius* var. *caesius* [74]. DOX has four anthraquinone rings with connection to one amino sugar moiety [75]. The first discovery towards antitumor activity of DOX returns back to 1950, but its application in the clinic was approved in 1963. The preferred administration route for DOX is intravenous with a recommendation dose of 50–75 mg·m^2^. DOX is administered in a single dose and repetition occurs after 21 days [75,76,77]. DOX interferes with cancer progression via intercalation with DNA and reducing activity of topoisomerase II, resulting in suppressing DNA replication. Although DOX demonstrated potential antitumor activity against different cancer cells, it was soon found that its efficiency in cancer therapy is restricted by a phenomenon known as chemoresistance.

To date, a high number of molecular pathways and mechanisms have been identified that contribute to DOX resistance. Trefoil factor 3 (TFF3) is a secreted protein with overexpression in small and large intestines and is correlated with enhanced cell migration [78]. Antiapoptotic and proproliferative functions of TFF3 are mediated by its trefoil domain and a cysteine residue (Cys057) in carboxy-terminal [79,80]. Following doxorubicin administration, an increase occurs in the expression of TFF3 and Akt that are responsible for cancer proliferation and inhibiting apoptosis. It has been reported that downregulation of TFF3 provides the way for enhancing apoptosis and doxorubicin sensitivity [81].

Uncontrolled proliferation of cancer cells is dependent on glucose uptake and glycolysis. Sphingosine kinase 1 (SphK1) accounts for triggering cancer chemoresistance and also, elevating glycolysis [82,83,84]. A newly published article has shed some light on the relationship between SphK1 and DOX resistance in osteosarcoma (OS) cells. Exposing OS cells to DOX is associated with upregulation of SphK1 and subsequent increase in glucose metabolism, leading to induction of chemoresistance [85].

Noteworthy, non-coding RNAs also play a significant role in DOX resistance. It seems that enhancing expression of oncosuppressor miRNAs such as miRNA-124 [86] and miRNA-223 [87] is of importance in impairing cancer proliferation and migration, and increasing their sensitivity into DOX chemotherapy. Furthermore, reducing activity of drug transporters such as P-gp elevates DOX accumulation in cancer cells and is of importance in reversing chemoresistance [88]. Overall, studies are in agreement with the fact that different molecular pathways and mechanisms can involve in inducing DOX chemoresistance and phytochemical are potential agents in triggering chemosensitivity [89,90,91,92].

However, chemoresistance is not the only drawback of DOX. A dose-dependent side effect is another challenge in DOX chemotherapy. Cardiotoxicity, hepatotoxicity and nephrotoxicity of major adverse effects of DOX. Stimulation of molecular pathways such as Akt that promote cell proliferation is of importance in reducing DOX side effects [93]. Non-coding RNAs such as miRNA-146a are able to prevent apoptotic cell death in improving DOX-mediated cardiotoxicity [94]. Noteworthy, plant derived-natural products have been extensively applied in improving DOX side effects. These agents enhance activity of antioxidant enzymes such as superoxide dismutase (SOD) and glutathione (GSH), while they diminish malondialdehyde (MDH) levels in preventing cardiotoxicity [95], through upregulation of Nrf2 signaling pathway [96].

**Figure 1:**
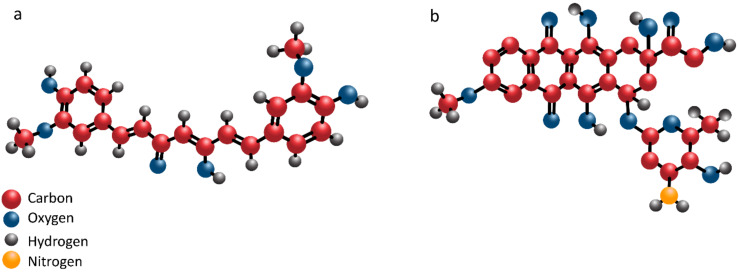
The chemical structure of curcumin (**a**) and doxorubicin (**b**).

In alleviation of DOX toxicity, phytochemicals reduce levels of proinflammatory cytokines via downregulating NF-κB signaling pathway [97]. Furthermore, apoptotic proteins such as Bax and caspase-3 undergo downregulation, while antiapoptotic factor Bcl-2 demonstrates an increase in expression to ameliorate DOX side effects [98]. Taking everything into together, studies demonstrate that inflammation, oxidative stress and apoptosis are induced following DOX exposure in normal cells and protective agents possess regulatory effect on aforementioned mechanisms in reducing adverse effects of DOX [99,100,101,102]. Figure 1 demonstrates the chemical structure of curcumin and doxorubicin.

## 6. Curcumin in Combination with Doxorubicin

### 6.1. Anticancer Effects

#### 6.1.1. Apoptosis Induction

Neuroblastoma is one of the common solid extracranial cancers in children that forms in tissues of the sympathetic nervous system [103]. Neuroblastoma is responsible for up to 15% of death in children [104]. The most important issue in neuroblastoma is the metastasis of these malignant cells. Up to 70% of neuroblastoma cells possesses metastatic capability and are able to involve bone, bone marrow and liver during migration [105,106]. Furthermore, they demonstrate uncontrolled growth. So, having a potential regimen for suppressing malignant behavior of neuroblastoma is recommended.

Coadministration of curcumin (5–50 μM) with DOX results in a decrease in viability and proliferation of neuroblastoma cells by apoptosis induction via upregulating p53 and p21. These effects are dose-dependent (with highest inhibitory effect at dose of 50 μM) [107]. Another highlights the fact that time is also important, so that a combination of curcumin (0–30 μg/mL) and DOX substantially induces apoptosis via Bcl-2 downregulation, and Bax, and caspase-9 upregulation in a time- and dose-dependent manner [108]. In induction of apoptosis during DOX chemotherapy, curcumin is able to affect other proapoptotic factors plus p53 and p21. Curcumin (100 mg/kg) promotes expression of caspase-3, -8 and -9 in potentiating antitumor activity of DOX [109].

The composition of gut microbiota determines the response to chemotherapeutic agents and checkpoint inhibitor therapies [110]. Gut microbiota is a key player in preserving mucosal barrier integrity and micronutrient levels such as Zn homeostasis. It has been reported that changes in Zn levels is correlated with emergence of HCC and a low potential of chemotherapy. Furthermore, clinical studies have revealed that patients with low levels of Zn demonstrate an increase in tumor size [111,112]. In order to meet these difficulties, curcumin-Zn solid dispersion (ZnCM-SD) has been developed for promoting efficacy of DOX in HCC therapy. ZnCM-SD is taken up by cells through non-specific endocytosis and is degraded in cells to Zn ions and curcumin. This combination synergistically suppresses cancer growth and viability and induces apoptosis in HCC cells. This is performed by preventing gut dysbiosis and regulating zinc homeostasis. This inhibitory effect of ZnCM-SD on HCC survival leads to enhanced efficacy of DOX in cancer chemotherapy [113].

#### 6.1.2. Metastasis Inhibition

Matrix metalloproteinase-2 is a determinant factor of cell migration. It enhances invasion of cancer cells via providing extracellular matrix (ECM) and basement membranes degradation [114,115]. Tissue inhibitors of metalloproteinases (TIMPs) are endogenous inhibitors of MMPs. TIMP-1 reduces expression of MMP-2 in impairing cancer migration [116,117]. Curcumin reduces migration of neuroblastoma cells and enhances their sensitivity to DOX chemotherapy by enhancing TIMP-1 expression and reducing MMP-2 expression [107].

Epithelial-to-mesenchymal transition (EMT) is a process in which epithelial cells acquire the mesenchymal phenotype [118]. Mesenchymal phenotypes possess stem cell features, increased generation of ECM components and enhanced cell migration [119,120,121,122]. Different molecular pathways act as upstream mediators of EMT in cancer progression. TGF-β signaling pathway is an EMT inducer in cancer cells and in this way, can provide chemoresistance [123,124]. PI3K/Akt signaling can also induce EMT in cancer cells, and its downregulation is associated with a decrease in EMT [125,126]. Noteworthy, curcumin has demonstrated capacity of affecting both aforementioned signaling networks in impairing EMT. Curcumin (20 μM) substantially promotes antitumor activity of DOX against triple-negative breast cancer cells by suppressing EMT and metastasis. In this way, curcumin downregulates expression of TGF-β and PI3K/Akt that are vital for EMT induction. Consequently, an increase occurs in E-cadherin levels, while N-cadherin levels demonstrate a decrease, paving the way for inhibiting EMT and enhancing antitumor activity of DOX [127].

Taking everything into account, studies are in agreement with the fact that curcumin is a potential adjuvant for polychemotherapy with DOX. It induces apoptosis in cancer cells via affecting caspase cascade and pro- and antiapoptotic proteins. Migration of cancer cells is disrupted by curcumin. Besides, gut dysbiosis is improved by curcumin in promoting efficacy of DOX in chemotherapy.

### 6.2. Effects on Resistance

#### 6.2.1. Potential Mechanisms of DOX Resistance

ATP-binding cassette (ABC) transporters are one of the most important factors involved in triggering chemoresistance. In addition to chemoresistance, ABC transporters contribute to material transportation and cellular homeostasis [128]. Most of the ABC transporters in eukaryotes are efflux transporters that provide elimination of chemotherapeutic agents from cells at the route of cytoplasm to extracellular space [128]. ABC sub-family B member 4 (ABCB4) has high sequence similarity with ABCB1 (up to 80%) and participates in chemoresistance [129]. PI3K acts as an upstream mediator of P-gp in cancer cells, and reducing its expression is correlated with downregulation of P-gp and chemosensitivity [130]. Notably, phytochemicals are potential agents in suppressing P-gp expression and promoting DOX sensitivity [131]. The interesting point is that activity of P-gp can be affected by other proteins in cancer cells. S100 calcium-binding protein A8 (S100A8) belongs to S100 multigene subfamilies and is correlated with cancer progression [132,133]. Accumulating data demonstrates role of S100A8 in chemoresistance [134].

#### 6.2.2. Curcumin in Reversing DOX Resistance

Curcumin administration (0–100 μM) enhances internalization and accumulation of DOX in breast cancer cells to improve its antitumor activity and suppress chemoresistance. It has been reported that this activity is mediated via reducing activity and expression of ABCB4, as a potential factor involved in pumping DOX out of breast cancer cells [135]. Inhibiting activity of P-gp is associated with mitotic slippage and apoptosis induction in multidrug resistance lung cancer cells [136]. In order to improve efficacy of curcumin in inhibiting P-gp activity upon DOX treatment, a combination of tannic acid and epigallocatechin gallate (EGCG) was used with curcumin. Results confirmed that leukemia cells are sensitive towards inhibitory effects of DOX after using a mixture of curcumin, EGCG and tannic acid. This is attributed to downregulation of P-gp that is of importance for enhancing intracellular accumulation of DOX in cancer cells [137].

**Figure 2:**
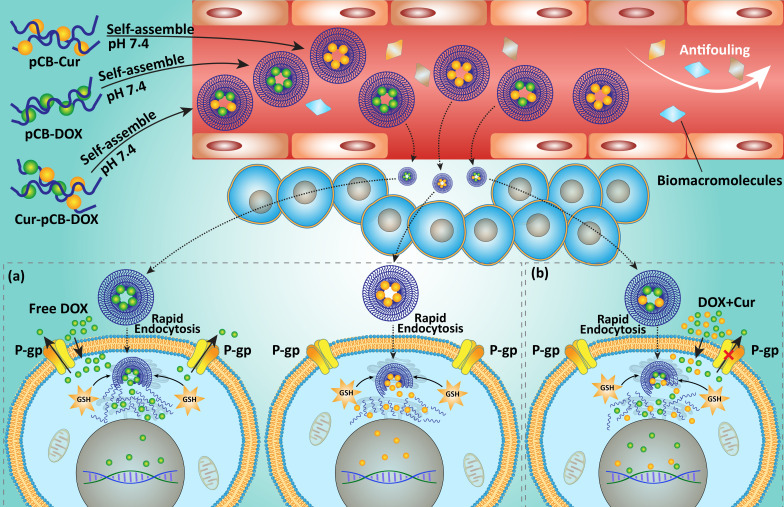
Schematic diagram of the proposed mechanism for promoting cellular uptake and overcoming MDR by Cur-pCB (poly(carboxybetaine)) -Dox in MCF-7/Adr cells. The nanosized feature of the zwitterionic antifouling micelles and endocytic pathway to bypass P-gp-mediated drug efflux led to high cellular uptake of the conjugated drugs that were released in the tumor cells of high GSH concentration. However, pCB-Dox and pCB-Cur can be delivered separately into different cells so that the drugs fail to play a synergistic role in inhibiting the MDR effect (**a**). By contrast, Cur-pCB-Dox codelivers Dox and Cur into the same tumor cells and results in synergistic effects of the two drugs (**b**). Merging images of cell uptake of pCB-Dox (left), pCB-Dox + pCB-Cur (middle) and Cur-pCB-Dox (right) in MFC-7/Adr cells (**c**). Cur: curcumin; Dox: doxorubicin; MDR: multidrug resistance; pCB: poly(carboxybetaine). Addapted with permission from [139].

In DOX-resistant leukemia cells, an increase occurs in expression of P-gp and S100A8. Downregulation of S100A8 enhances sensitivity of cancer cells to DOX chemotherapy. The increased sensitivity of cancer cells to DOX chemotherapy upon S100A8 inhibition is not due to enhanced accumulation of DOX, but also due to enhanced intracellular free calcium ion content and overexpression of proapoptotic proteins. Curcumin (0–32 μM) as a potential antitumor agent, reduces expression of S100A8 and P-gp in a time- and dose-dependent manner to promote DOX accumulation and induce apoptosis in leukemia cells [138]. These studies demonstrate that drug transporters participate in DOX resistance and they are potential targets of curcumin in providing chemosensitivity. As an example, it was shown that colocal delivery of DOX and curcumin can synergistically increase the effectiveness toward multidrug-resistant cancer cells, e.g., further reducing the *IC*_50_ value by 37% (8.9 μg/mL; Figure 2) [139].

Increasing evidence demonstrates the role of NF-kB and activator protein-1 (AP-1) in triggering chemoresistance [140,141]. These two molecular pathways are potential targets of curcumin in reversing chemoresistance. It is held that curcumin (0–50 μmol/L) reduces expression of AP-1 and NF-kB via downregulation of Akt and JNK. Negative regulation of these signaling networks is vital for induction of apoptosis by curcumin in glioblastoma cells and providing chemosensitivity. In enhancing sensitivity to doxorubicin chemotherapy, curcumin also reduces expression of DNA repair enzymes such as MGMT, DNA-PK, ERCC-1, Ku70 and Ku80 to potentiate DNA damage [142].

Noteworthy, curcumin derivatives have also demonstrated great capability in reversing DOX resistance. HER2 is suggested to be involved in chemoresistance [143]. The exact mechanism in which HER2 induces chemoresistance has not been understood. A recent study has shown that downregulation of miRNA-196a-5p occurs during chemoresistance that provides HER2 overexpression [144]. Curcumin and its derivatives (PGV-0 and PGV-1) downregulate expression of NF-kB and HER2 to induce cell cycle arrest at the G1 phase, leading to enhanced sensitivity of breast cancer cells to DOX chemotherapy [145].

**Figure 3:**
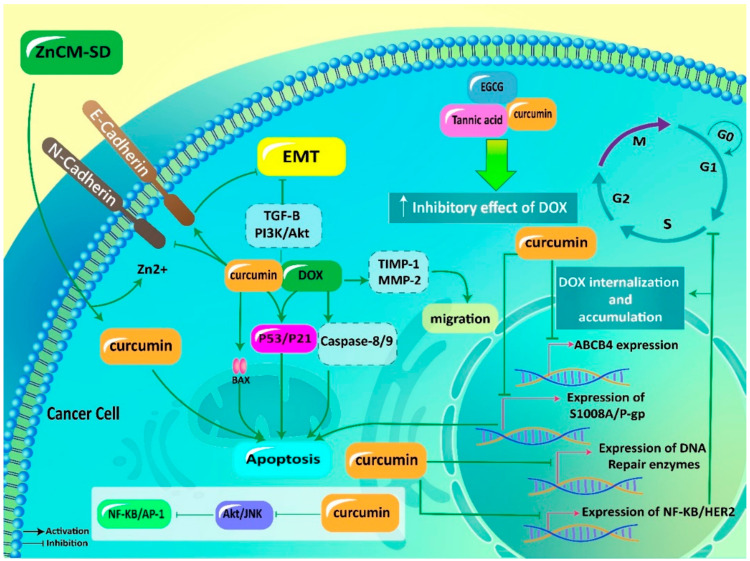
Curcumin in promoting antitumor activity of DOX and reversing chemoresistance. ZnCM-SD, curcumin-Zn solid dispersion; EMT, epithelial-to-mesenchymal transition; TGF-β, transforming growth factor-beta; PI3K, phosphatidylinositol 3-kinase; Akt, protein kinase B; DOX, doxorubicin; TIMP-1, tissue inhibitor of matrix metalloproteinase-1; MMP-2, matrix metalloproteinase-2; EGCG, epigallocatechin gallate; AP-1, activator protein-1; NF-κB, nuclear factor-kappaB; P-gp, P-glycoprotein.

Using nanoparticles such as micelles remarkably promotes intracellular uptake of curcumin and DOX, leading to more efficiency in suppressing chemoresistance compared to DOX or curcumin alone [139]. The ideology of coadministration of curcumin and DOX during cancer chemotherapy was better advocated, when it was found that DOX is also capable of promoting cellular uptake of curcumin in enhancing its antitumor activity against prostate cancer cells [146]. This study demonstrates dual relationship between curcumin and DOX in cancer cells, urging scientists for their coadministration in providing effective cancer chemotherapy (Figure 3, Table 1) [147].

These studies highlight the fact that P-gp and ABCB4 are able to induce DOX resistance by preventing DOX accumulation in cancer cells. Impairing activity and expression of P-gp and ABCB4 by curcumin results in enhanced internalization of DOX in cancer cells and providing chemosensitivity. Notably, signaling pathways that promote cancer progression including NF-κB, AP-1 and PI3K/Akt, are inhibited by curcumin to suppress cancer proliferation, resulting in an increase in DOX sensitivity.

### 6.3. Impact on Adverse Effects

The clinical application of DOX is limited due to its dose-dependent cardiotoxicity. Oxidative stress, iron-loading disorders, calcium dysregulation, inflammation and apoptosis contribute to DOX-mediated cardiomyopathy [149]. Noteworthy, gut microbiota plays a significant role in the regulation of chemotherapy-mediated toxicity [150,151,152]. Gut dysbiosis is a common finding in cancer [153]. Studies have shown the importance of gut microbiota in DOX toxicity. It seems that gut microbiota depletion leads to a decrease in DOX-mediated toxicity in heart, kidney, liver and intestine [154,155]. Following DOX exposure, gut dysbiosis occurs that may lead to disruption in the intestinal barrier. An impairment occurs in heart function and cardiomyocytes undergo apoptosis. A combination of curcumin and Zn improves DOX-mediated gut dysbiosis via enhancing the number of beneficial bacteria including *Clostridium*_XIVa, *Clostridium*_IV, *Roseburia*, *Butyricicoccus* and *Akkermansia*. This combination also maintains intestine barrier integrity and prevents DOX-mediated zinc dyshomeostasis. Apoptosis in cardiomyocytes is inhibited and an improvement occurs in heart function [156].

The pathogenesis of DOX-mediated cardiotoxicity has been completely understood. However, studies demonstrate the potential role of inflammation, oxidative stress and apoptosis in triggering cardiotoxicity following DOX chemotherapy [157]. Curcumin administration (100 and 200 mg/kg) enhances activities of antioxidant enzymes such as SOD, GSH and catalase (CAT) in reducing oxidative stress. Besides, curcumin inhibits inflammation via downregulation of NF-κB, tumor necrosis factor-alpha (TNF-α) and interleukin-1β (IL-1β). A decrease occurs in apoptosis following curcumin administration by caspase-3 downregulation. Furthermore, DNA damage is prevented. These protective effects lead to an alleviation in DOX-mediated cardiotoxicity [158].

It is worth mentioning that curcumin can be coadministered with other naturally occurring compounds in improving its capacity in ameliorating DOX-mediated cardiotoxicity. Carvacrol (CAR) is a phenol compound present in essential oils of plant species such as *Origanum*, *Thymus* and *Coridothymus*. CAR has demonstrated potential cardioprotective effects. For instance, it can ameliorate diabetic cardiomyopathy via triggering PI3K/Akt pathway via PTEN downregulation [159]. Furthermore, it is beneficial in improving pathologic cardiac hypertrophy [160]. A combination of curcumin (100 mg/kg) and CAR (50 mg/kg) is advantageous in protecting cardiomyocytes against DOX toxicity. This combination heals the heart function and reduces the volume of connective tissues and cardiomyocytes. Furthermore, curcumin and CAR promote the volume of myocardium and vessels, and number of cardiomyocyte nuclei in preventing DOX-mediated toxicity [161].

Mitochondrial phosphate carrier (PiC) contributes to importing phosphate across inner membrane of mitochondria [162]. The role of PiC in releasing cytochrome C from mitochondria, activating caspase cascade and apoptosis induction has been confirmed [163]. Increased levels of PiC are associated with cell death, but decreasing its levels is correlated with a decrease in apoptosis [162]. Curcumin uses a same strategy in protecting against DOX-mediated cardiotoxicity. Curcumin (10, 12 and 15 mg/L) prevents cardiomyocyte apoptosis following DOX chemotherapy. In this way, curcumin reduces mRNA levels of PiC, as a major pathway for inhibiting apoptosis. Furthermore, curcumin diminishes oxidative stress to prevent mitochondrial dysfunction and subsequent apoptosis in cardiomyocytes [164]. It seems that maintaining mitochondrial function via reducing ROS levels is the major way that is followed by curcumin in preventing DOX-mediated cardiotoxicity [165].

Notably, cardioprotective activity of curcumin against DOX toxicity can be improved using nanoparticles. Mesoporous silica nanoparticles (MSNs) significantly promote bioavailability of curcumin that is beneficial for decreasing oxidative damage and reinforcing the antioxidant defense system by enhancing activities of SOD, CAT and GSH [166]. According to the fact that poor bioavailability of curcumin is a barrier for its therapeutic effects, loading curcumin on nanoparticles remarkably promotes its bioavailability that is of importance for preventing DOX-mediated cardiotoxicity [167]. It is held that enhancing ROS overgeneration by DOX leads to the stimulation of mitochondrial pathway of apoptosis. Due to possessing high antioxidant activity, curcumin reduces levels of ROS in preventing DOX-mediated cardiotoxicity.

It was mentioned that curcumin exerts its cardioprotective effect in a dose- and time-dependent manner. Mode of treatment is also a determining factor in protective effects of curcumin. Simultaneous treatment of cardiomyocytes with curcumin and DOX potentiates cardiotoxicity of DOX, while pretreatment with curcumin is of importance in repressing DOX-mediated cardiotoxicity [168]. So, time, dose and mode of treatment are important for cardioprotective activity of curcumin and using nanoparticles significantly promotes therapeutic efficacy of curcumin [167].

Previously, we demonstrated that heart is the main target of DOX. Heart function is disrupted after DOX chemotherapy, and apoptosis occurs in cardiomyocytes. However, heart is not the only target of DOX. It has been shown that testis is negatively affected by DOX during chemotherapy. The same pathways are followed for providing DOX toxicity on testis including inflammation, oxidative stress and apoptosis. Curcumin administration (100 and 200 mg/kg) for 7 days significantly promotes sperm motility and the number of live sperms. It decreases MDH levels and improves the antioxidant condition. Necrosis, degeneration, slimming in seminiferous tubules and DNA damage are prevented following curcumin administration. These protective effects of curcumin against DOX-mediated testis toxicity are dose-dependent with the highest protective effect at dose of 200 mg/kg [169].

Kidney is also negatively affected by DOX in chemotherapy. Tubular atrophy and enhanced glomerular capillary permeability occurs following DOX chemotherapy [170]. ROS are considered as the main factors in this process [171]. Curcumin administration (100 and 200 mg/kg) for 7 days decreases oxidative damage in kidney via promoting activity of antioxidant enzymes such as SOD, CAT and GSH. Inflammation is inhibited by curcumin via downregulation of TNF-α, NF-κB, IL-1β, COX-2 and iNOS. Furthermore, curcumin prevents DNA damage and suppresses apoptosis through reducing expressions of capase-3 [172]. Nephrotic syndrome (NS) emanates from severe proteinuria due to edema and intravascular volume depletion [173]. A recent study has revealed that DOX is able to induce NS in rats. Consequently, curcumin has been applied for alleviation of this pathological condition. It seems that curcumin (100 and 200 mg/kg) is a promising candidate in amelioration of DOX-mediated NS. Curcumin attenuates proteinuria and is beneficial in improving hypoalbuminemia. Curcumin also reduces lipid levels to ameliorate hyperlipidemia in rats [174]. As it was mentioned before, inflammation and oxidative stress are two major pathways for providing DOX-mediated nephrotoxicity. NF-κB participated in inflammation [175] and curcumin downregulates its expression in improving NS [174].

**Figure 4:**
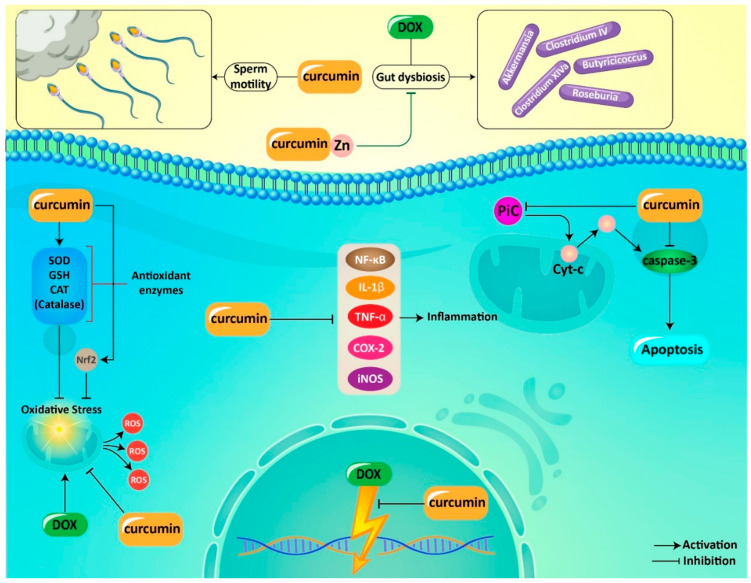
Curcumin in alleviation of adverse effects of DOX during chemotherapy. DOX, doxorubicin; cyt c, cytochrome C; NF-κB, nuclear factor-kappa B; IL-1β, interleukin-1β; TNF-α, tumor necrosis factor-a; COX-2, cyclooxygenase-2; iNOS, inducible nitric oxide synthase; SOD, superoxide dismutase; GSH, glutathione; CAT, catalase; ROS, reactive oxygen species; PiC, mitochondrial phosphate carrier.

Oxidative stress is ameliorated by upregulation of Nrf2 signaling pathway, as a factor involved in enhancing expression of antioxidant enzymes such as heme oxygenase-1 (HO-1) and NAD(P)H dehydrogenase [quinone] 1 (NQO1) [176,177]. By upregulation of Nrf2, curcumin reinforces the antioxidant defense system, resulting in alleviation of NS [174]. Conducted experiments related to protective effects of curcumin against DOX-mediated cardiotoxicity demonstrate that inflammation, oxidative stress and apoptosis are the major mechanisms responsible for cardiotoxicity of DOX. Due to possessing antioxidant and anti-inflammatory activities, curcumin can effectively alleviate DOX-mediated cardiotoxicity. Further studies can focus on evaluating protective effects of curcumin on other side effects of DOX such as hepatotoxicity, nephrotoxicity and neurotoxicity (Figure 4, Table 2).

## 7. Codelivery via Biological Nanovehicles

It is worth mentioning that both DOX and curcumin suffer from poor bioavailability. In light of this, a wide variety of nanoplatforms have been developed for codelivery of curcumin and DOX [179,180,181]. In addition to promoting cellular internalization of DOX, nanocarriers can prevent development of chemoresistance, since a low amount of DOX is loaded on/in the encapsulants. Thus, it is quite obvious that using nanostructures is beneficial for polychemotherapy of curcumin and DOX. This section highlights the application of various nanosized carriers for codelivery of curcumin and DOX in cancer therapy.

Previous studies have confirmed the potential of lipid nanoparticles for delivery of curcumin [182,183] and DOX [184,185] due to their outstanding features including biocompatibility, high encapsulation efficiency (EE), prolonged drug release, stability, targeted delivery and enhancing antitumor activity. Notably, lipid nanocarriers are also beneficial for codelivery of curcumin and DOX in different sorts of cancers, e.g., liver cancers. Lipid-based nanomaterials offer sustained release of curcumin and DOX that significantly enhances their antitumor activity. In vivo experiment also showed that curcumin- and DOX-loaded lipid NPs are capable of suppressing cancer growth [186]. It has been reported that curcumin- and DOX-loaded lipid NPs have more capability in apoptosis induction via enhancing caspase-3 expression and Bax/Bcl-2 ratio compared to curcumin and DOX alone [187].

**Figure 5:**
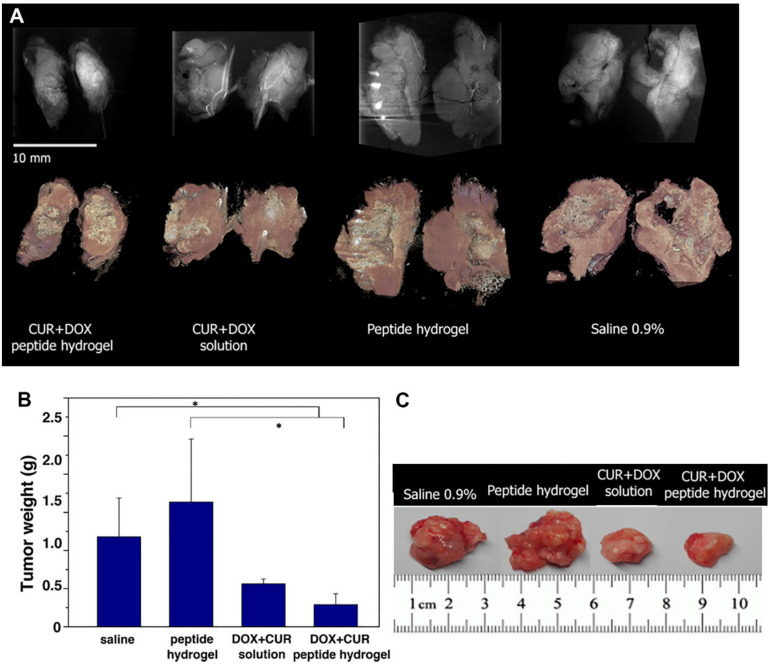
(**A**) Side-by-side comparison of the tumors studied by μCT. Top row: Summation of intensity along the stack of CT slices (*n* slices ≈400) showing density variations and overall dimensions of the tumors. Bottom row: 3D photorealistic rendering of the tumors. (**B**) Tumor weight (* *p* < 0.05 vs. control and blank treatment groups). (**C**) Representative images of tumors excised at the end point of the study on day 15. Reprinted with permission from [194], ACS Publications, 2019.

The c-Myc is an oncogene pathway in promoting proliferation and invasion of cancer cells via interacting with other molecular pathways such as SOX4 [188]. The c-Myc signaling pathway is inhibited by curcumin- and DOX-loaded nanoparticles in HCC therapy [187]. A prerequisite for metastasis of cancer cells is angiogenesis induction. Vascular endothelial growth factor (VEGF) participates in cancer progression and migration via angiogenesis induction [189]. Downregulation of VEGF and P-gp occurs following exposing HCC cells to curcumin- and DOX-loaded lipid nanoparticles [187]. Self-assembled micelles are another option in codelivery of DOX and curcumin in cancer therapy. Polymeric micelles have a core–shell structure to entrap lipophilic active compounds in their hydrophobic core to enhance drug circulation in blood as results of their hydrophilic shell. Notably, polymeric micelles can selectively accumulate in cancer cells via enhanced permeability and retention (EPR) effect [190,191]. Polymeric micelles are able to enhance the cellular uptake of DOX through energy-dependent and caveolae-mediated endocytosis. These nanoparticles are able to suppress multidrug resistance (MDR) via CD44 targeting. Codelivery of curcumin and DOX by polymeric micelles significantly induces apoptosis in breast cancer cells [192]. Micelles not only can be applied for codelivery of curcumin and DOX, and enhancing their antitumor activity, but also can be applied for reducing DOX-mediated cardiotoxicity [193] Although cancer therapy is the main focus of this review, the aforementioned study provides the fact that micelles are potential nanocarriers for promoting bioavailability of curcumin and improving its therapeutic effects. In another study, self-assembly was employed for the local codelivery via peptide-based hydrogel, targeting head and neck cancer (Figure 5). The synergistic pharmacological effects were observed in codelivery of DOX and curcumin [194].

However, more progress can be made in promoting the efficacy of nanovehicles in curcumin and DOX delivery. In this regard, surface functionalization of micelles can be a good strategy. For instance, surface decorating with antibodies that selectively target overexpressed receptors on cancer cells is of importance. In light of this, glucose transporter-1 (GLUT-1), which is the responsible antibody for glucose uptake in cells, is a promising option [195]. Why is that? Although GLUT-1 is expressed on all kind of normal cells, its expression is substantially increased in cancer cells [196]. This is due to the need of cancer cells to glucose in providing enough energy for the Warburg effect [197]. Besides, GLUT-1 is distributed on the blood–brain barrier (BBB) plasma membrane and has overexpression on glioblastoma cells [198,199]. In order to selectively target glioblastoma cells, micelles have been modified with the GLUT-1 antibody. This is also of importance for promoting penetration of micelles via BBB. Overall, GLUT-1-modified micelles significantly elevate nuclear localization of DOX, and codelivery by curcumin has a synergistic effect. Apoptosis is induced by upregulation of caspase-3 and capase-7, and deep penetration into cancer cells occurs due to surface modification of micelles with GLUT-1 [200].

The tumor microenvironment plays a remarkable role in cancer progression. Enhanced levels of Th1 cells are associated with poor prognosis [201]. Nanomaterials can sense the tumor microenvironment for effective cancer therapy [202]. For instance, curcumin- and DOX-loaded liposomes can affect the Th1/Th2 axis in the tumor microenvironment for colon cancer therapy. Furthermore, apoptosis is induced by these nanocarriers and a decrease occurs in the migration of cancer cells [203]. Protein- and peptide-based nanostructures are also able to encapsulate small molecules with selective targeting of tumor cells and tissues [204,205,206,207].

NCA (α-amino acid-*N*-carboxyanhydrides and α-amino acid) ring-opening polymerization is an affordable method in nanotechnology [208]. The fabricated polyamino acid by this method is biodegradable and can undergo metabolism in a normal metabolic way that is of importance for its biocompatibility [208]. Recently, polypeptide nanocarriers have been applied for codelivery of curcumin and DOX in lymphoma treatment. Curcumin and DOX demonstrate a synergistic effect in apoptosis induction in lymphoma cells and using polypeptide nanocarriers significantly promotes their efficiency in apoptosis stimulation. Furthermore, curcumin- and DOX-loaded polypeptide nanocarriers were able to exert effect on non-coding RNAs in lymphoma therapy. Curcumin- and DOX-loaded polypeptide nanocarriers significantly reduce expression of oncogene miRNAs such as miRNA-21 and miRNA-199a, while they increase expression of oncosuppressor miRNAs including miRNA-98 and miRNA-200c [209].

One of the features of the tumor microenvironment is its mild pH. This is beneficial for designing smart nanocarriers that can release curcumin and DOX in tumor site (pH = 5) [210,211]. Furthermore, nanoparticles can be designed in selective targeting of receptors that undergo upregulation in cancer cells. Urokinase plasminogen activator receptor (uPAR) demonstrates overexpression in different cancer cells [212,213,214]. Recently, a smart pH-sensitive decorated with U11 peptide has been developed for targeted delivery of curcumin and DOX in lung cancer treatment. This nanocarrier is able to selective target lung cancer cells that overexpress uPAR. Furthermore, it releases curcumin and DOX in the pH of 5 that is similar to the pH of the tumor microenvironment. These excellent features lead to an increase in tumor accumulation of curcumin and DOX, and high cytotoxicity against cancer cells [215].

Stimuli-responsive prodrug nanoparticles have opened a new window in the production of smart and potential drug delivery systems. In this method, a small drug molecule is conjugated to a macromolecule. During circulation in blood, prodrug remains at its inactive form, while it is activated at the tumor site [216,217]. The pH-sensitive prodrug nanoparticles have been designed for codelivery of curcumin and DOX in cancer therapy. These antitumor-loaded nanoparticles demonstrate sustained circulation in blood and promote penetration to cancer cells. This increase in local drug accumulation results in enhanced antitumor activity of curcumin and DOX against cancer cells [218].

Noteworthy, nanocarriers not only promote antitumor activity of curcumin and DOX against cancer cells, but also are beneficial in preventing chemoresistance [219]. To effectively suppress the metastasis of cancer cells, delivery of curcumin and DOX to tumor vasculatures is of importance. This leads to inhibition of angiogenesis and disrupting cancer invasion. RGDK-lipopeptide is suggested to be a promising candidate for selective targeting of tumor vasculature [220]. For this reason, liposomes have been modified with RGDK-lipopeptide. These nanocarriers are able to deliver curcumin and DOX to tumor vasculature and demonstrate high cellular uptake due to the presence of RGDK-lipopeptide. After accumulation in cancer cells, curcumin and DOX suppress tumor progression via the downregulating TGF-b signaling pathway [221]. This study demonstrates how surface modification of nanoparticle is in favor of promoting cell internalization of curcumin and DOX and enhancing their antitumor activity.

**Figure 6:**
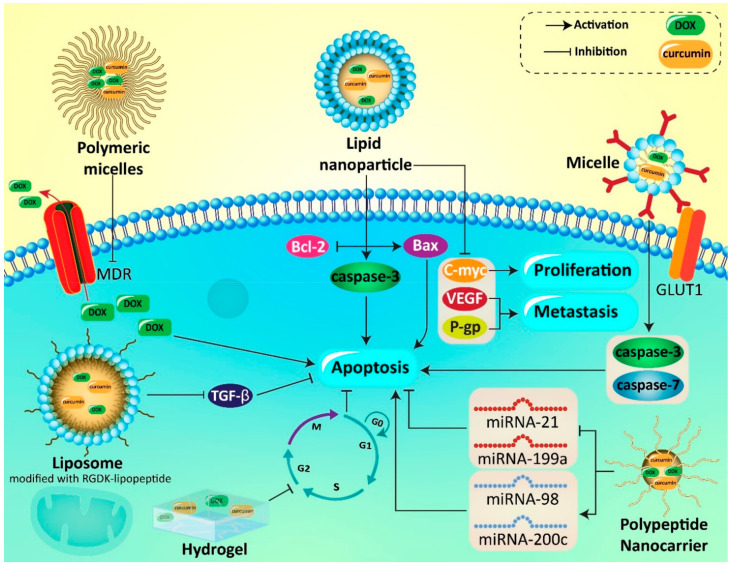
Curcumin- and DOX-loaded nanovehicles in effective cancer chemotherapy. DOX, doxorubicin; GLUT1, glucose transporter 1; VEGF, vascular endothelial growth factor; P-gp, P-glycoprotein; miRNA, microRNA; MDR, multidrug resistance; TGF-β, transforming growth factor-β.

As curcumin suffers from poor bioavailability and DOX has adverse effects, using nanocarriers can provide targeted delivery of these active compounds associated with an increase in their effectiveness Intracellular accumulation of DOX and curcumin enhances after using nanoencapsulant for their codelivery. The chemoresistance is prevented, since nanoparticles provide sustained release of curcumin and DOX. Smart nanomaterials (e.g., pH-sensitive and redox-sensitive nanoparticles) can be applied for targeted delivery of curcumin and DOX, to release their cargo in response to the tumor microenvironment. Besides, the surface of nanoparticles can be modified by receptors and ligands (such as hyaluronic acid and RGD) to promote accumulation in cancer cells (Figure 6) [222,223,224,225,226,227,228,229,230,231,232,233,234]. In Table 3, different nanocarriers applied for codelivery of curcumin and DOX are described. Besides, encapsulation efficiency, zeta potential and major outcomes of the experiments are provided in Table 3 to shed some light on the application of nanostructures for DOX and curcumin codelivery.

## 8. Conclusions and Remarks

Curcumin can be coapplied with DOX in promoting its antitumor activity. Curcumin potentiates the antitumor activity of DOX in a time- and dose-dependent manner. In enhancing the antitumor activity of DOX, curcumin upregulates expression of Bax, caspase-9, p53 and p21, while it reduces expression of Bcl-2 to stimulate apoptosis and promote the efficacy of DOX in chemotherapy. Besides, the metastasis of cancer cells is also negatively affected by curcumin in promoting DOX efficacy via MMP-2 downregulation and TIMP-1 upregulation. Curcumin inhibits EMT via downregulating TGF-β and PI3K/Akt signaling networks, leading to an increase in sensitivity of cancer cells to DOX chemotherapy.

Notably, in potentiating antitumor activity of DOX, curcumin can be coadministered with Zn to improve gut dysbiosis, resulting in an enhancement in efficacy of DOX in chemotherapy. The drug transporters (P-gp) are inhibited by curcumin in promoting DOX sensitivity. In addition to drug transporters, molecular pathways that are responsible for cancer progression, are affected by curcumin in reversing DOX resistance. Akt, JNK, AP-1 and NF-κB are among the oncogene pathways that are downregulated by curcumin. Besides, curcumin reduces expression of DNA repair enzymes including MGMT, DNA-PK, ERCC-1, Ku70 and Ku80 in promoting DOX sensitivity.

A variety of experiments have evaluated protective effects of curcumin against DOX-mediated cardiotoxicity. Gut dysbiosis occurs following DOX administration and curcumin improves this condition to provide proper function of heart. Furthermore, oxidative stress, inflammation and DNA fragmentation and apoptosis are prevented by curcumin in alleviation of DOX-mediated cardiotoxicity.

Application of NPs significantly enhances curcumin bioavailability and its therapeutic effects ameliorating DOX-mediated cardiotoxicity. Noteworthy, according to poor bioavailability of curcumin and DOX, nanomaterials such as polymeric NPs, liposomes, micelles and nanogels and inorganic nanostructures (e.g., metal/metal oxide nanocompounds) have been designed for codelivery of DOX and curcumin. These nanocarriers significantly promote cell internalization of DOX and curcumin, resulting in improved antitumor activity. Furthermore, smart NPs such as pH-sensitive ones have been designed for drug release at the tumor microenvironment. This review article revealed that curcumin is a potential adjuvant for polychemotherapy with DOX, and based on the safety of curcumin, clinical studies can begin evaluating efficiency of this regime in the treatment of cancer patients.

## Figures and Tables

**Table 1:** Curcumin as an enhancer of antitumor activity upon doxorubicin chemotherapy.

Cancer Type	In vitro/In vivo	Cell Line/Animal Model	Effect on Doxorubicin Efficacy	Dose	Experiment Duration	Remarks	Refs
Glioblastoma	In vitro	T98G (world health organization (WHO) grade IV), U87MG (WHO grade III), and T67 (WHO grade III) human glioma and C6 rat glioma cell lines	Enhancement	20, 40 and 60 μmol/L	48 h	Promoting inhibitory effect of DOX on cancer cells Reversing chemoresistance Downregulation of AP-1 and NF-κB Apoptosis induction Inhibiting JNK and Akt signaling pathways	[142]
Breast cancer	In vitro	MCF-7 cells	Enhancement	6-110 μM	24 h	Downregulation of HER2 and NF-κB Subsequent sensitivity into DOX chemotherapy	[145]
Breast cancer	In vitro	MCF-7 and MDA-MB-231 cells	Enhancement	0-100 μM	72 h	Downregulation of ABCB4 Increasing DOX accumulation and cytotoxicity	[135]
Chronic myeloid leukemia	In vitro	Human CML cell lines K562 and K562/DOX	Enhancement	0.5, 1 and 2 μM	48 h	Promoting sensitivity into DOX chemotherapy via P-gp and S100A8 downregulation	[138]
Hepatic cancer	In vitro	HA22T/VGH cells	Enhancement	10, 15, 20 and 25 μM	72 h	Downregulation of c-Myc and COX-2 Decreasing expression of Bcl-2, Bcl-Xl and c-IAP-2 levels Apoptosis induction	[147]
Colon cancer Breast cancer	In vitro	Human colon HCT116 and breast cancer MCF7 cell lines	Enhancement	15 μM	-	Apoptosis induction Exerting antiproliferative activity Enhancing DOX efficacy	[148]
Neuroblastoma	In vitro	SH-SY5Y cells	Enhancement	5-50 μM	24 h	Disrupting cancer invasion Downregulation of MMP-2 Upregulation of TIMP-1 Enhancing DOX sensitivity	[107]
Gastric adenocarcinoma	In vitro	AGS cells	Enhancement	0-30 μg/mL	24, 48, 72 and 96 h	Apoptosis stimulation via Bcl-2 downregulation and Bax and caspase-9 upregulation Suppressing tumor spheroid formation, proliferation and metastasis	[108]

**Table 2:** Alleviation of adverse effects of doxorubicin by curcumin.

In vitro/In vivo	Cell Line/Animal Model	Dose	Experiment Duration	Administration Route	Remarks	Refs
In vivo	Wistar rats	100 and 200 mg/kg	7 days	Oral gavage	Reducing oxidative stress and MDH levels Promoting sperm motility and viability Preventing necrosis and degeneration	[169]
In vivo	Rat	100 and 200 mg/kg	7 days	Oral administration	Preventing apoptosis via caspase-3 downregulation Ameliorating inflammation via TNF-α and NF-κB downregulation Inhibiting DNA damage	[172]
In vivo	DOX-mediated cardiotoxicity in rat	100 mg/kg of curcumin 50 mg/kg CAR	22 days	Intraperitoneal administration	Reducing volume of myocardium and vessels Decreasing number of cardiomyocyte nuclei Improving heart function Decreasing connective tissue volume	[161]
In vivo	Rat	100 and 200 mg/kg	7 days	Oral administration	Alleviation of oxidative stress, inflammation, apoptosis and DNA damage Downregulation of TNF-α and NF-κB	[158]
In vivo	Rat	100 and 200 mg/kg of curcumin 1 and 2 mg/kg of nebivolol	30 days	Intraperitoneal administration	Enhancing survival rate Improving body weight, heart index and ECG parameters Preventing oxidative damage and apoptosis	[178]

**Table 3:** Codelivery of curcumin and doxorubicin in providing effective cancer chemotherapy.

Nanocarrier	In vitro/In vivo	Cell Line/Animal Model	Encapsulation Efficiency (%)	Particle Size (nm)	Zeta Potential (mV)	Remarks	Refs
Liposome	In vitro	B16F10 cells	100 (DOX) 86 (curcumin)	190–230	2–4	Surface modification of liposomes with RGDK promotes their cellular uptake Enhancing antitumor activity by 2–3 folds	[221]
Polymeric nanoparticles	In vitro	HepG 2 and HeLa cells	18.35 (DOX) 91 (curcumin)	183.5	−0.68	Possessing pH sensitivity capability Being inactive at blood circulation Activation at tumor site Enhancing antitumor activity	[218]
Polymeric nanoparticles	In vitro	HUVEC cells and MCF-7/ADR cells	92	115–135	0.41	High drug loading Hemodynamic stability Intracellular accumulation Suppressing cancer progression	[235]
Polymeric nanoparticles	In vitro In vivo	NIH-3T3 (mouse embryonic fibroblast cells), HeLa (human cervical cancer cells), NCI-H460 (Human lung carcinoma cells), and HFL1 (Human normal lung cells)	23–53	180–220	10–15	Apoptosis stimulation in resistant cancer cells DNA fragmentation Enhancing bioavailability and antitumor activity	[236]
Micelle	In vitro In vivo	H9C2 cells tumor-bearing mice	90.6–99.8	90.6–120	−0.13 to −2.34	High encapsulation efficiency and drug loading Sustained drug release Elevating DOX accumulation in cancer cells Providing a delay in cancer growth	[193]
Micelle	In vitro	U87MG cells	-	14.4 to 14.8	−4.2 to −4.4	Apoptosis induction via caspase-3 and caspase-7 upregulation Deep internalization via GLUT-1 Decreasing cancer viability	[200]
Polymeric micelle	In vitro	HepG2 and HUVEC cells	90.9 (DOX) 70.7 (curcumin)	80–110	−0.5 to +10	Redox-responsive drug release Internalization through endocytosis Providing a synergistic effect and enhancing antitumor activity	[237]
Polymeric micelles	In vitro	MCF-7 cells	-	164.2–190	-	Enhanced cellular uptake due to EPR effect Elevating cytotoxicity against cancer cells	[139]
Micelle	In vitro	MDA-MB-231 cells	94.69 (DOX) 99.97 (curcumin)	60	−16.4	Exerting synergistic effect Apoptosis induction	[238]
Selenium nanoparticles	In vitro In vivo	HCT116 cells Tumor-bearing mice	-	202–240	−31 to −37	Increasing ROS levels Disrupting mitochondrial homeostasis Triggering apoptosis and cell cycle arrest Suppressing metastasis via EMT and NF-κB downregulation Autophagy inhibition Promoting antitumor activity	[239]
Solid lipid nanoparticles	In vivo	Hodgkin’s lymphoma in mice	-	125.2	−19.4	Promoting curcumin bioavailability Reducing cancer growth and viability Apoptosis induction via XIAP and Mcl-1 downregulation Ameliorating inflammation by reducing pro-inflammatory cytokines including IL-6 and TNF-α	[240]
Solid lipid nanoparticles	In vitro In vivo	Human MCF-7 cells, human TNBC MDA-MB-231 and murine mammary cancer JC cells Tumor xenograft	-	-	-	Enhancing cytotoxicity against cancer cells by 5-10 folds Inhibition of NF-κB signaling pathway and subsequent decrease in P-gp expression Increasing accumulation of antitumor agents in cancer cells	[241]
Mesoporous silica nanoparticles	In vitro	MCF-7 cells	-	-	-	Localization in cytoplasmic vesicles Triggering apoptosis via capase-6, -9 and -12 upregulation Activation of PTEN and CHOP for stimulating apoptosis Disrupting mitochondrial homeostasis Autophagy induction by nanoparticles	[242]

## Abbreviations

ROS	reactive oxygen species
EMT	epithelial-to-mesenchymal transition
miRNA	microRNA
PI3K	phosphatidylinositol 3-kinase
Akt	protein kinase-B
NF-κB	nuclear factor-kappaB
HCC	hepatocellular carcinoma
P-gp	P-glycoprotein
MDR1	multidrug resistance 1
Nrf2	nuclear factor erythroid 2-related factor 2
TGF-β1	transforming growth factor-beta 1
DOX	doxorubicin
TFF3	trefoil factor 3
SphK1	sphingosine kinase 1
OS	osteosarcoma
GSH	glutathione
SOD	superoxide dismutase
MDH	malondialdehyde
ECM	extracellular matrix
TIMPs	tissue inhibitors of metalloproteinases
EMT	epithelial-to-mesenchymal transition
ZnCM-SD	curcumin-Zn solid dispersion
ABC	ATP-binding cassette
ABCB4	ABC sub-family B member 4
EGCG	epigallocatechin gallate
S100A8	S100 calcium-binding protein A8
AP-1	activator protein-1
CAT	catalase
TNF-α	tumor necrosis factor-α
IL-1β	interleukin-1β
CAR	carvacrol
PiC	mitochondrial phosphate carrier
MSNs	mesoporous silica nanoparticles
EE	encapsulation efficiency
VEGF	vascular endothelial growth factor
EPR	enhanced permeability and retention
MDR	multidrug resistance
GLUT-1	glucose transporter-1
BBB	blood-brain barrier
uPAR	urokinase plasminogen activator receptor
HO-1	heme oxygenase-1
NQO1	NAD(P)H dehydrogenase [quinone] 1
